# Mannitol Challenge to Assess Therapy Response in Asthmatic Children: An Interventional Cohort Study

**DOI:** 10.3390/children10050802

**Published:** 2023-04-28

**Authors:** Nikolaos Karantaglis, Fotios Kirvassilis, Elpis Hatziagorou, Antonios Gkantaras, Kalliopi Kontouli, John Tsanakas, Maria Emporiadou

**Affiliations:** 1 Pediatric Pulmonology and Cystic Fibrosis Unit, 3rd Pediatric Department, Aristotle University of Thessaloniki, Hippokration General Hospital of Thessaloniki, Konstantinoupoleos Str. 49, 54642 Thessaloniki, Greece; fkirvassilis@gmail.com (F.K.); hatziagorou@auth.gr (E.H.); kkontoul@otenet.gr (K.K.); tsanakasj@gmail.com (J.T.); 2 Pediatric Immunology and Rheumatology Referral Center, 1st Department of Pediatrics, Aristotle University of Thessaloniki, Hippokration General Hospital of Thessaloniki, Konstantinoupoleos Str. 49, 54642 Thessaloniki, Greece; agkantar@auth.gr; 3 School of Medicine, Faculty of Health Sciences, Aristotle University of Thessaloniki, 54124 Thessaloniki, Greece; eboriad@auth.gr

**Keywords:** pediatric asthma, mannitol challenge, bronchial hyperresponsiveness, bronchial provocation tests, asthma prophylaxis

## Abstract

Bronchial provocation tests, such as the mannitol challenge, can be performed to identify and quantify the severity of bronchial hyperresponsiveness in asthmatic patients. Studies of the mannitol challenge as a monitoring tool in asthmatic children are limited. Our primary aim was to compare the bronchial hyperresponsiveness to mannitol in treatment-naive asthmatic children between baseline and three months after receiving the indicated asthma prophylaxis. Twenty-three asthmatic patients aged 4–16 years were analyzed in this prospective cohort study. All subjects underwent the mannitol challenge at baseline and after three months of treatment with budesonide ± formoterol. The difference in the provocative dose of mannitol to induce a 15% drop in FEV_1_ (PD_15_) between baseline and follow-up, as well as its association with the presence of exercise-induced or nocturnal asthma symptoms, were evaluated. The PD_15_ value increased significantly post-treatment (228.5 mg [4.50–458.15]; *p* = 0.04). Independently of the evaluation time point, the PD_15_ values were significantly lower in the presence of nocturnal asthma symptoms (490 mg [122–635] vs. 635 mg [635–635]; *p* = 0.03), whereas there was no association between the PD_15_ value and the presence of exercise-induced asthma (*p* = 0.73). These results suggest that bronchial hyperresponsiveness to mannitol may be a potential monitoring tool in the pediatric asthmatic population, reflecting therapy response in children receiving prophylactic treatment.

## 1. Introduction

Asthma is the most common chronic disease of childhood, with increasing prevalence worldwide during the last five decades and is imposing a major burden on global public health systems [1,2]. The pathophysiology of this chronic respiratory disease involves intermittent obstruction of the airflow due to bronchial inflammation and airway hyperresponsiveness to direct or indirect stimuli. Both these pathophysiologic features of asthma can be present even in patients with mild disease and/or normal lung function, leading eventually to severe exacerbations with a significant negative impact on the quality of life of asthmatic children and adolescents [3,4,5]. Asthma prophylaxis with inhaled corticosteroids (ICS) combined or not with long-acting β2-agonists (LABAs) has been proven effective to reverse and prevent airway inflammation and bronchial hyperresponsiveness (BHR), improving health outcomes in asthmatic subjects [3,6].

Taking into account the clinical heterogeneity of childhood asthma, the definitive diagnosis remains challenging, requiring a comprehensive review of current and past medical history, family history and asthma triggers, as well as a thorough physical examination. The clinical diagnosis of asthma—based on the presence of typical symptoms, such as recurrent wheezing, cough, shortness of breath or chest tightness, especially during exertion or nocturnal sleep—is supported by pulmonary function testing through spirometry, which demonstrates a reversible obstructive pattern [7,8]. However, a non-negligible proportion of pediatric asthmatic patients with active airway inflammation retain normal pulmonary function on presentation, necessitating the performance of bronchial provocation tests (BPTs) to identify objectively and quantify the severity of BHR, further establishing the diagnosis of asthma [3].

Depending on the underlying mechanism of bronchoconstriction, BPTs are divided into direct and indirect ones. During direct BPTs, bronchoconstriction is induced by the administration of pharmacological agents such as methacholine or histamine, acting directly on specific bronchial smooth muscle cell receptors, whereas indirect challenges utilize either physical stimuli (exercise or eucapnic hyperpnea) or osmotic agents (mannitol or hypertonic saline) to induce bronchoconstriction “indirectly” through the release of bronchospastic and inflammatory mediators from airway immune cells [9]. Whereas direct BPTs are highly sensitive, presenting a high negative predictive value to exclude an asthma diagnosis in the absence of BHR to either methacholine or histamine, they are not absolutely specific for asthma-associated BHR. On the other hand, indirect challenges, such as mannitol, are characterized by their high specificity in establishing an asthma diagnosis, even in patients with negative responses to direct constricting agents. It has to be highlighted that indirect BPTs are considered more asthma-specific, as they mimic the natural stimuli evoking bronchoconstriction in asthmatic subjects, tending to correlate better with the persistence and the extent of airway inflammation compared to direct BPTs [10,11]. So, indirect BPTs, such as a mannitol dry powder (MDP) challenge, may be used to identify asthmatic individuals most likely to benefit from prophylactic ICS [9,12].

The MDP challenge has been demonstrated as a practical tool for BHR evaluation in clinical practice due to the combination of portability, reproducibility and high specificity in asthma diagnosis [13,14,15]. In addition, it has been suggested as a potential monitoring tool for assessing the impact of ICS prophylaxis on airway inflammation and BHR in asthmatic subjects [12,16]. On this basis, it has been proposed that a negative response to an MDP challenge may indicate an optimal therapeutic response in asthmatic patients receiving ICS [17]. Nevertheless, studies of the MDP challenge as a monitoring tool in asthmatic children and adolescents are limited and heterogeneous, evaluating either the outcome of stepping down asthma prophylaxis in pretreated subjects or the effect of a rather short-term prophylaxis treatment [18,19].

The primary aim of the current study was to compare the BHR response to mannitol in ICS-naive asthmatic children and adolescents between baseline and three months after receiving asthma prophylaxis. The secondary aims were: (a) to investigate the association between response to an MDP challenge and the presence of exercise-induced or nocturnal asthma symptoms and (b) to confirm the tolerability of the MDP challenge in our study group.

## 2. Materials and Methods

### 2.1. Subjects and Ethical Permission

Children and adolescents aged 4–16 years old who had been referred for the first time to the outpatient clinic of the Pediatric Pulmonology Unit of the Third Department of Pediatrics of the Aristotle University of Thessaloniki (AUTh) due to recurrent episodes of physician-diagnosed wheezing and respiratory distress during the previous two years, indicating high suspicion of lower airway obstruction and bronchial hyperresponsiveness (BHR), were evaluated. According to the GINA guidelines, subjects with a clinical diagnosis of asthma, based on the history or presence of wheezing, dyspnea, chest tightness or cough, especially during exercise or nocturnal sleep, were screened for trial eligibility [3]. Among them, ICS-naive patients (i.e., who had never been on long-term anti-asthmatic prophylaxis) without underlying comorbidities or other concurrent chronic respiratory diseases were asked to participate in this study. Parents of eligible subjects agreed to participate in the study and signed consent forms after having the aims and the procedures involved in the study explained thoroughly. The study protocol was approved by the Bioethics Committee of the Medical School of AUTh (Ref No. 2655). All patient data was collected and treated according to the Declaration of Helsinki.

### 2.2. Study Design

This prospective, open-label cohort study comprised two visits. During the first visit, all patients had their medical history, as well as their demographic and somatometric data, recorded. In addition, they underwent a thorough clinical evaluation and spirometry testing (Ergoline, Vmax Series V.20-1, SensorMedics, Anaheim, CA, USA) to assess baseline pulmonary function. Subsequently, the mannitol dry powder (MDP) challenge was performed according to the manufacturer’s protocol using the corresponding commercially available kit (Aridol^®^, Pharmaxis, Frenchs Forest, Sydney, Australia).

All participants were prescribed ICS ± LABA according to the GINA guidelines [3]. Moreover, they were asked to return for a scheduled follow-up visit three months after treatment initiation. A written plan of instructions and information on rescue and controller medications was given, and the proper inhalation technique was demonstrated so as to ensure optimal treatment effects.

During the follow-up visit, all patients were evaluated clinically and underwent the MDP challenge. Treatment was adjusted for each patient accordingly, and an appointment for reevaluation was arranged.

### 2.3. Mannitol Dry Powder Challenge

All patients were required to withhold the use of short-acting beta2-agonists (SABAs) for 8 h before each MDP challenge test and the use of LABAs and ICS for 24 h before the second MDP challenge, as recommended by the manufacturer [12].

The MDP challenge was conducted according to the protocol [20]. Briefly, capsules containing escalating doses of mannitol (0, 5, 10, 20, 40, 2 × 40, 4 × 40, 4 × 40, and 4 × 40 mg) were administered consecutively in nine steps to each patient via an inhaler device. After the inhalation of each dose, spirometry was performed, and forced expiratory volume in one second (FEV_1_) was recorded. The FEV_1_ measurement after the administration of the placebo capsule (0 mg) was used as the baseline measurement. Unless all nine steps were completed successfully and a cumulative dose of 635 mg mannitol was administered, the challenge was terminated when a 15% drop in FEV_1_ in relation to the baseline was recorded, or a 10% fall in FEV_1_ occurred between two consecutive doses. In these cases, the response to the MDP challenge was considered positive. For all subjects, the provocative dose of mannitol to induce a 15% drop in FEV_1_ (PD_15_) and the response–dose–ratio (RDR = percentage of maximum drop in FEV_1_/maximum dose mannitol administered) were calculated using the software provided by the manufacturer (PD_15_ Calculator for Aridol^®^ Bronchial Challenge Test Kit). In children who did not complete the challenge, PD_15_ was calculated by linear interpolation of the relationship between the percent drop in FEV_1_ from baseline at the termination of the MDP challenge and the cumulative dose of mannitol to be administered to induce this drop. If PD_15_ was calculated equal to or greater than 635 mg, the response to the MDP challenge was considered negative. After the end of the challenge, each patient received 300 mcg of salbutamol with a spacer and performed a final spirometry to ensure the restoration of normal airflow 15 min later.

### 2.4. Statistical Analysis

Continuous variables were presented as mean (standard deviation [SD]) for parametric data and as median (25th–75th interquartile range [IQR]) for non-parametric data, whereas categorical dichotomous variables were expressed as absolute (*n*) and relative (%) frequencies. Comparisons between continuous variables before and after intervention were performed using the two-sided paired *t*-test or Wilcoxon signed rank test for parametric and non-parametric data, respectively. McNemar’s test was used to compare dichotomous variables between baseline and follow-up. Univariate and multivariate linear regression models were applied to evaluate the influence of body mass index (BMI) *Z*-score, clinical (exercise-induced asthma and nocturnal asthma), and spirometric (FEV_1_% predicted) parameters on PD_15_, both at baseline and follow-up. The significance level (α) was set to 0.05 for all analyses. Data management and statistical analyses were performed using the R programming language v4.1.3 [21].

## 3. Results

### 3.1. Subjects

**Figure 1:**
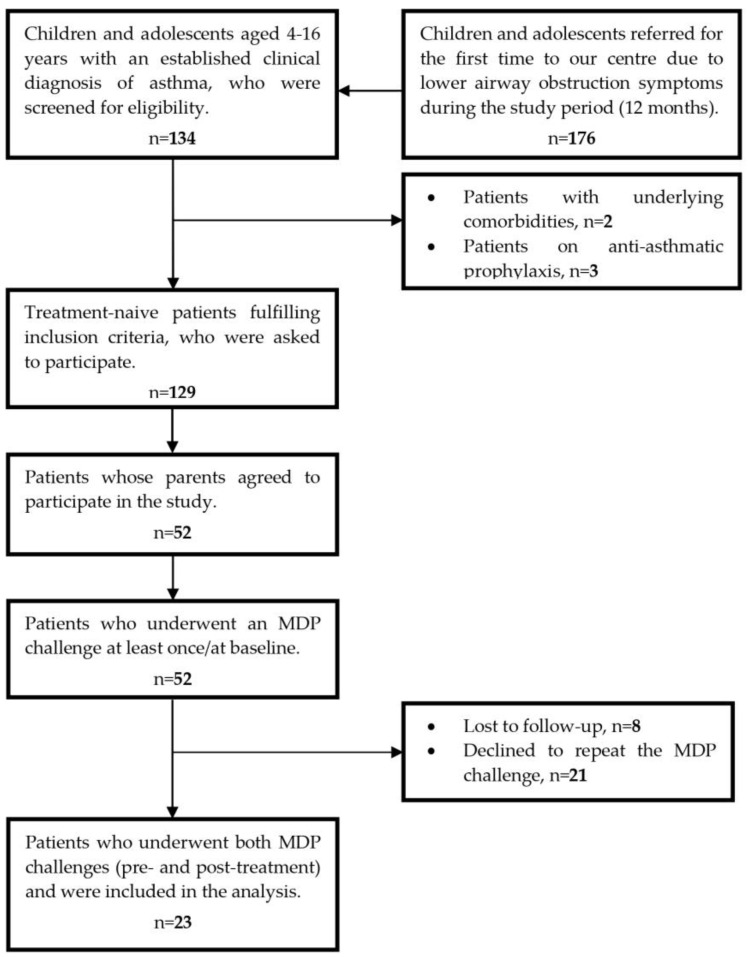
Flow diagram showing the recruitment process and study completion by participants.

A total of 52 subjects with a clinical diagnosis of asthma (Male: Female = 38: 14), aged 9.69 ± 2.17 years, were enrolled in the study and underwent the MDP challenge at baseline before treatment initiation. Of these, 44 subjects showed up for the scheduled follow-up evaluation, whereas 21/44 participants (47.72%) expressed unwillingness to perform the MDP challenge at the follow-up visit due to the experienced discomfort (nausea/tendency to vomit: *n* = 15; mucosal burning sensation: *n* = 4; dizziness: *n* = 2) during the initial test. Overall, 23 subjects completed the study, having undergone the MDP challenge at both evaluation time points and were included in the analysis. An overview of the recruitment process, as well as the progress of participants from enrollment to study completion, is depicted in Figure 1. Baseline demographic and clinical data of patients are included in the analysis and presented in Table 1.

### 3.2. Effect of Asthma Prophylaxis on Clinical Outcomes and BHR to Mannitol

After three months of asthma prophylaxis, all participants had normal lung function with a mean FEV_1_ of 107.14% (±12.56%) predicted, which was not statistically different from baseline FEV_1_ (113.93% ± 17.10% predicted; *p* = 0.20). Post-treatment, there was a significant decrease in the frequency of nocturnal symptoms (4.35% vs. 56.52% at baseline; *p* < 0.01), whereas the percentage of patients reporting exercise-induced symptoms decreased slightly (56.52% vs. 69.57% at baseline; *p* = 0.26).

**Figure 2:**
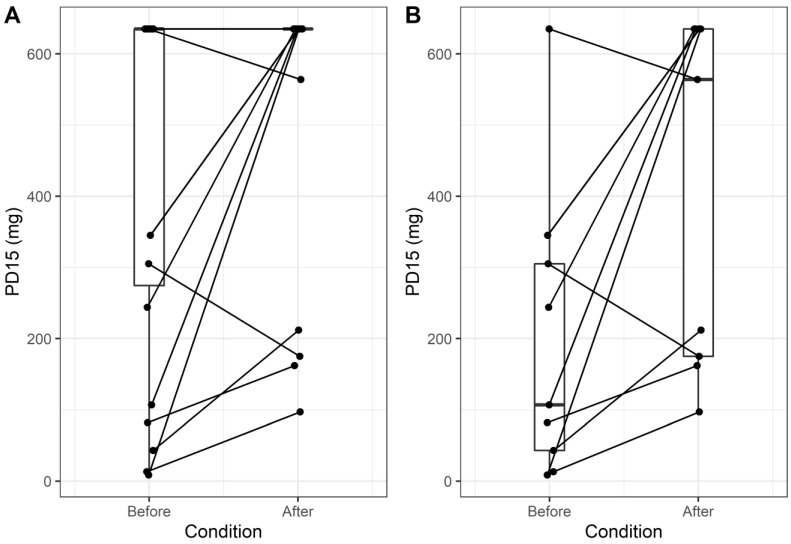
Paired box plots depicting changes in PD_15_ before and after treatment for all patients of the cohort (**A**); and after exclusion of patients with a negative MDP challenge at both evaluation time points (**B**).

During the first visit, 8/23 children demonstrated a positive response to the MDP challenge. Of these, four maintained their positive response during the follow-up visit despite the prophylactic treatment, whereas the rest (4/8; 50%) became unresponsive to mannitol (Figure 2A). Of note, one patient had a negative response to the MDP challenge at baseline and developed airway hyperresponsiveness to mannitol in the post-treatment evaluation. The PD_15_ increased significantly post-treatment (pseudo-median difference 228.5 mg; 95% CI 4.50 to 458.15; *p* = 0.04), whereas a significant decrease was demonstrated in RDR (pseudo-median difference −1.22%/mg; 95% CI −2.85 to −0.20; *p* = 0.04). Furthermore, 14/23 (60.87%) participants had a PD_15_ ≥ 635 mg in both pre- and-post-treatment evaluation. Of the remaining nine patients with a change in PD_15_ over the follow-up period, only two had lower PD_15_ at three months after treatment as compared to baseline (Figure 2B).

Changes in all studied outcome parameters between the two evaluation time points are shown in Table 2.

### 3.3. Associations between PD_15_ and Patient Characteristics

The results of the regression analysis are shown in Table 3. At baseline, PD_15_ values were significantly associated with the presence of nocturnal asthma symptoms (*p* = 0.01) and BMI-for-age *Z*-score (*p* < 0.01). Post-treatment, no significant association was observed between PD_15_ and the presence of nocturnal asthma symptoms, which is justified with only a single patient reporting symptoms during nocturnal sleep at follow-up.

**Figure 3:**
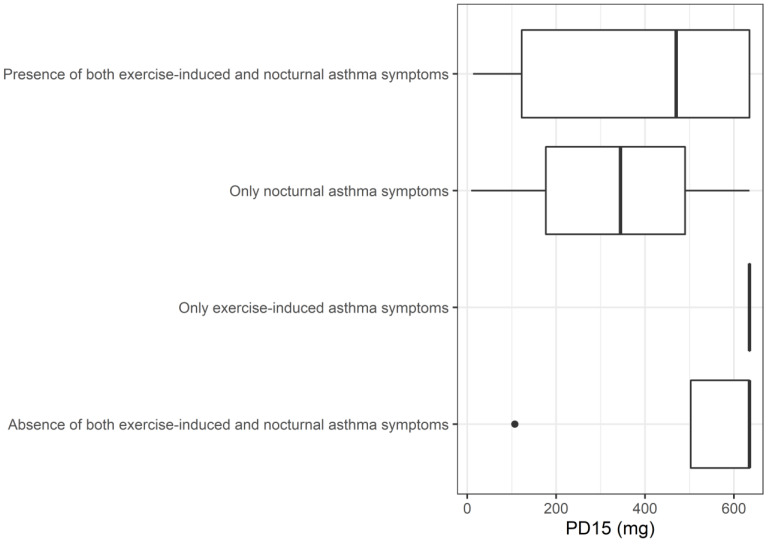
Box plots depicting the distribution of the PD_15_ value among the clinical groups. The PD_15_ at baseline tended to be lower in the presence of nocturnal asthma symptoms that were either accompanied or not by the presence of exercise-induced asthma.

Taking into account the possible overlap between exercise-induced and nocturnal asthma, we distributed our patients into four distinct groups, according to their clinical symptoms at baseline: Group 1: “Presence of both exercise-induced and nocturnal asthma symptoms” (*n* = 10); Group 2: “Only nocturnal asthma symptoms” (*n* = 3); Group 3: “Only exercise-induced asthma symptoms” (*n* = 6); and Group 4: “Absence of both exercise-induced and nocturnal asthma symptoms” (*n* = 4). Despite the fact that the Kruskal–Wallis test did not demonstrate any significant difference in PD_15_ between these groups (*p* = 0.15), children experiencing nocturnal symptoms (Group 1 and 2) tended to have lower PD_15_ [Group 1: 470 mg (122–635); Group 2: 345 mg (177–490); Group 3: 635 mg (635–635); Group 4: 635 mg (503–635)], as depicted in Figure 3.

**Figure 4:**
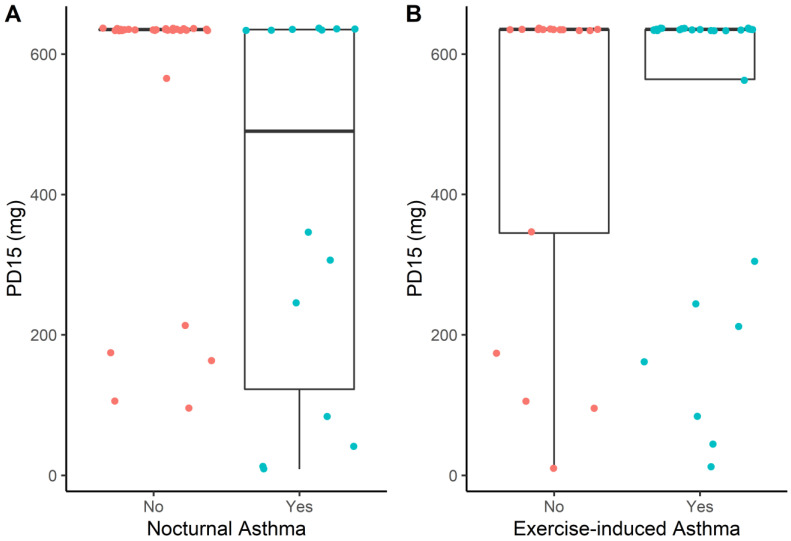
Box plots depicting the association between the PD_15_ value and the existence of nocturnal asthma symptoms (**A**) or exercise-induced asthma (**B**). The PD_15_ was significantly lower when patients reported asthma-related symptoms during nocturnal sleep (*p* = 0.03), whereas it was not affected by the presence of exercise-induced asthma (*p* = 0.73).

Independently of the evaluation time point, the PD_15_ values were significantly lower in the presence of nocturnal asthma symptoms [490 mg (122–635) vs. 635 mg (635–635); *p* = 0.03], whereas there was no significant difference in PD_15_ regarding the presence of exercise-induced asthma [635 mg (564–635) vs. 635 mg (302–635); *p* = 0.73] (Figure 4).

### 3.4. Safety, Tolerability and Adverse Events of the MDP Challenge

No patient experienced a serious adverse event attributable to the MDP challenge. However, during the first visit, 11/52 patients (21.15%) asked to discontinue the test: eight patients reported discomfort due to nausea and/or tendency to vomit, one complained of a burning sensation in the larynx, while the remaining two could not cooperate satisfactorily. On the other hand, during the second visit, 3/23 patients (13.04%) asked to terminate the challenge prematurely due to severe general discomfort and/or an urgent tendency to vomit. Despite the fact that an occasional cough was noted in the majority of subjects, especially during the administration of higher doses of mannitol, severe coughing, leading to the disruption of the challenge, occurred in no cases. It has to be highlighted that none of the participants exhibited severe dyspnea, requiring oxygen supplementation, during the trial. All subjects with a positive MDP challenge response fully recovered after receiving a single dose of 300 mcg salbutamol, as documented by both a clinical and spirometric evaluation after 15 min. All participants were discharged in a stable condition.

## 4. Discussion

In this study, we investigated the effect of asthma prophylaxis on BHR to mannitol in ICS-naive asthmatic children and adolescents. We found that three months of prophylaxis with the indicated—according to GINA guidelines [3] and the internal protocol of our center’s anti-asthmatic agent resulted in a significant decrease of BHR to mannitol, as reflected by the corresponding increase in the post-treatment PD_15_ value. The observed difference in PD_15_ between the two evaluation time points should be attributed to the changes in patients with a positive MDP challenge at baseline. Under this prism, the response to the MDP challenge in ICS-naive patients with a clinical diagnosis of asthma may be an additional tool in clinical decision-making, indicating who would benefit the most from asthma prophylaxis.

Interestingly, in two patients, a decrease in the PD_15_ value was demonstrated post-treatment with combination therapy. This finding could be interpreted either by inadequate compliance or the reported effects of LABA on BHR. In an attempt to explain similar findings, previous studies have hypothesized that long-term concurrent treatment with LABAs leads to the development of bronchodilator tolerance due to the downregulation and desensitization of the target β2 adrenergic receptor, especially on mast cells [18,22].

Regarding clinical outcomes, a significant reduction of nocturnal asthma symptoms was demonstrated after three months of treatment. In our study, the improvement of nocturnal asthma symptoms, which is a significant indicator of effective disease control, was significantly associated with the attenuation of BHR following proper anti-asthmatic treatment. Specifically, the absence of nocturnal asthma symptoms was associated with higher PD_15_ values independently of the effect of other confounders, such as the BMI *Z*-score, the FEV_1_ predictive value and the presence of exercise-induced asthma. These findings suggest that BHR to mannitol may be potentially used to monitor response to anti-asthmatic therapy, quantifying disease control simultaneously through the PD_15_ value. To our knowledge, this is the first study demonstrating a correlation in pediatric asthmatic patients between BHR to mannitol and the presence of a particular clinical feature of asthma, namely, nocturnal symptoms. Previous studies have demonstrated a positive correlation between the presence of nocturnal symptoms in asthmatic patients and the levels of exhaled or alveolar nitric oxide (NO) [23,24]. In addition, increased levels of exhaled NO have been associated with lower PD_15_ values and increased BHR to mannitol, reflecting ongoing airway inflammation [25,26]. Therefore, it becomes evident that the aforementioned studies cooperatively provide support for our results, highlighting the role of PD_15_ as a potential candidate biomarker of the presence of nocturnal symptoms in asthmatic children.

However, our study did not find an association between the response to the mannitol test and the presence of symptoms during exercise, despite the fact that several previous studies report the mannitol test as a useful test for detecting patients with a positive exercise test [27,28]. However, it should be noted that our patients were selected based on the clinical diagnosis of asthma, while the aforementioned studies recruited patients with a positive exercise test or exercise-induced bronchoconstriction [19].

Our study confirms the favorable safety profile of the MDP challenge in the pediatric population, as evidenced by the absence of serious adverse events. This is in accordance with previous studies evaluating the safety and feasibility of the MDP challenge in asthmatic children and adolescents [13,27,29]. Even in subjects who developed wheezing during the MDP challenge, it completely resolved after the administration of a single dose of 300 mcg salbutamol, which was reflected by the return of FEV_1_ to baseline levels within 15 min of the completion or termination of the MDP challenge. Although we did not evaluate the precise recovery time following the administration of salbutamol, the 15-min recovery time in our sample was in the range of the previously reported times (1–20 min), being sufficient for complete restoration of normal lung function without the need for a second dose of salbutamol [18]. In accordance with the literature, none of the participants experienced a persistent cough severe enough to prevent them from completing the MDP challenge [9]. Nevertheless, in a non-negligible percentage of patients, the MDP challenge was discontinued due to reported general discomfort, overwhelming nausea or a burning sensation in the larynx. These adverse events have also been recorded in previous studies, in addition to headaches, which were not observed in our patients [9,12,28]. It has to be highlighted that nausea and the burning mucosal sensation were the main reasons for the unwillingness to repeat the MDP challenge at the follow-up visit. In our study, the relatively high percentage of patients refusing to undergo a second MDP challenge calls into question the reported high tolerability [22,27,30,31].

Of note, the consecutive maneuvers of the MDP challenge should be performed quickly without prolonged interruptions, as delaying the administration of mannitol could result in milder osmotic stimuli and, consequently, false negative responses [22,32]. Except for coughs that interfere with the timely execution of the consecutive steps of the MDP challenge [22], triboelectrification of the inhaler and the capsules has been described as a cause hindering the seamless delivery of consecutive mannitol doses [33]. A similar phenomenon of the capsule not properly spinning in the corresponding chamber of the device or even attached to the walls of the inhaler was observed in our study. However, we attributed this to humidity rather than triboelectrification, as the capsules were moist as a consequence of the patients repeatedly breathing in and out.

The major strength of the current study is its design, which reflects the daily clinical practice in a pediatric respiratory outpatient clinic by including newly diagnosed treatment-naive asthmatic patients and by evaluating treatment outcomes after adequate treatment duration (3 months) following the GINA guidelines. All participants were first-time referrals for clinical evaluation and treatment who had never been on long-term anti-asthmatic prophylaxis prior to their initial visit. Moreover, the selection of the study population was based mainly on the clinical diagnosis of asthma after a thorough history and physical examination, as well as pulmonary function testing during the initial visit. These factors contrast with previous similar clinical studies, which evaluated BHR to mannitol either in asthmatic children on inhaled corticosteroids [18], or in children with exercise-induced bronchoconstriction and a positive exercise challenge test at baseline [19]. In contrast to the short follow-up period of previous reports (two to four weeks), our study investigated the effect of asthma prophylaxis on BHR to mannitol in asthmatic children and adolescents after a long and adequate follow-up period (three months) [18,19]. In addition, the vast majority of the existing literature has focused more on the diagnostic potential of the MDP challenge, assessing its sensitivity and specificity, compared to other direct and indirect BPTs, in identifying asthmatic children and adolescents, rather than its potential role as a monitoring tool in pediatric asthma follow-up [13,26,27,28,31,32,33,34,35].

We should also consider the limitations of the current study. This study is a single-center, non-randomized, open-label prospective cohort study characterized by the absence of a control group. In addition, the sample size is relatively small, though comparable to relevant published studies [18,19]. Nevertheless, we believe that these limitations also present future research opportunities.

## 5. Conclusions

Our study confirms the safety of the MDP challenge in asthmatic children, albeit many will experience non-serious adverse events (e.g., discomfort, nausea, etc.) and may not complete the procedure. Nevertheless, a negative test correlates with the absence of nocturnal asthma symptoms independently of the level of lung function, thus suggesting that the MDP challenge may be used as a complementary tool for assessing the response to anti-asthmatic treatment, even in outpatient settings.

## Figures and Tables

**Table 1:** Baseline demographic and clinical characteristics of the study population (*n* = 23).

	Mean (SD)
Age (years)	9.98 (2.10)
Height (cm)	143.26 (12.44)
Weight (kg)	42.22 (13.33) *
BMI *Z*-score	1.25 (1.46)
FEV_1_ (% predicted)	110.66 (19.15)
	**N (%)**
Sex (Male)	16 (69.57)
Exercise-induced asthma symptoms	16 (69.57)
Nocturnal asthma symptoms	13 (56.52)
Prophylactic treatment-naive	23 (100.00)
Initiated asthma prophylaxis	
ICS	2 (8.70)
ICS + LABA	21 (91.30)

* Weight was not normally distributed; the corresponding median (IQR) was 45.00 (30.00–51.50).

**Table 2:** Comparison of mannitol challenge test results and clinical characteristics in our study sample (*n* = 23) before and after intervention.

	Pre-Treatment	Post-Treatment	Difference (95% CI)	*p*-Value
PD_15_ (mg) *	635.00 (259.25–635.00)	635.00 (635.00–635.00)	228.50 (4.50, 458.15)	0.04
RDR (mg/mL)	1.70 (0.50–3.20)	0.70 (0.30–0.90)	−1.22 (−2.85, −0.20)	0.02
FEV_1_ (% predicted)	113.93 (17.10)	107.14 (12.56)	−6.01 (−15.50, 3.48)	0.20
Exercise-induced asthma symptoms	16 (69.57)	13 (56.52)		0.26
Nocturnal asthma symptoms	13 (56.52)	1 (4.35)		<0.01
Completed mannitol challenge	16 (69.57)	20 (86.96)		0.16
Positive mannitol challenge **	8 (34.78)	5 (21.74)		0.18

The PD_15_ and RDR are expressed as median (IQR) (non-parametrical variables), FEV_1_ is expressed as mean (SD) (parametrical variable), and the categorical variables are expressed as the number of cases (%). * The mean (SD) of pre- and post-treatment PD_15_ was 464.04 (251.14) and 549.57 (183.51), respectively. ** In children who did not complete the challenge, PD_15_ was calculated by linear interpolation of the relationship between the percent drop in FEV_1_ from baseline at the termination of the MDP challenge and the cumulative dose of mannitol to be administered to induce this drop. If PD_15_ was calculated as greater than 635 mg, the response to the MDP challenge was considered negative.

**Table 3:** Results of the regression analysis using the PD_15_ value as a dependent variable in each evaluation time point.

		Univariate Analysis		Multivariate Analysis	
	Independent Variable	Coefficient (95% CI)	*p*	Coefficient (95% CI)	*p*
Pre-treatment (adj. R^2^ = 0.41)	BMI-for-age (Z-score)	89.92 (23.62 to 156.21)	0.01	92.10 (33.61 to 150.59)	<0.01
	Nocturnal Asthma (Yes)	−209.05 (−412.91 to −5.20)	0.04	−216.78 (−385.73 to −47.83)	0.01
	Exercise-induced Asthma (Yes)	50.84 (−190.30 to 291.99)	0.67	Not included *	
	FEV_1_ (% predicted)	−2.26 (−8.84 to 4.32)	0.48	Not included *	
Post-treatment (adj. R^2^ = 0.22)	BMI-for-age (Z-score)	63.91 (14.98 to 112.85)	0.01	63.91 (14.98 to 112.85)	0.01
	Nocturnal Asthma (Yes)	89.32 (−308.01 to 486.64)	0.64	Not included *	
	Exercise-induced Asthma (Yes)	25.42 (−138.48 to 189.31)	0.75	Not included *	
	FEV_1_ (% predicted)	−0.18 (−7.82 to 7.47)	0.96	Not included *	

* In the multivariate analysis, only the variables with *p*-values < 0.2 in the univariate analysis were included.

## Data Availability

Data available on request.
